# Digital Tools for Boosting the Impact of Fiscal Decentralization in Africa's Local Economies

**DOI:** 10.12688/f1000research.143922.1

**Published:** 2024-04-16

**Authors:** Nara Monkam, Maonei Gladys Mangwanya

**Affiliations:** 1 Department of Economics, University of Pretoria, Pretoria, Gauteng, 0028, South Africa

**Keywords:** Fiscal decentralization, Property tax, Subnational government finance, Local economic development, Digital technologies

## Abstract

**Background:**

Fiscal decentralization offers potential for stimulating local economic development in Africa, empowering subnational governments to control revenue and spending. However, challenges such as urbanization, poverty, inequality, insufficient infrastructure, and governance issues hinder the successful implementation of the core tenets of fiscal decentralization. This paper explores the role of digital technologies in promoting greater fiscal decentralization and revenue enhancement, as a strategic response to these local economic development challenges in Africa.

**Method:**

Through a systematic literature review, this study explores the role of fiscal decentralization in driving local economic development, with a focus on leveraging digital technologies to boost revenue generation and strengthen governance and institutional capacity at the subnational level.

**Results:**

The research underscores the importance of investing in digital infrastructure, skill development, and regulatory frameworks, while addressing data privacy and security concerns.

**Conclusion:**

By emphasizing the transformative impact of digital technologies fiscal decentralization and property taxation, this paper contributes to the existing literature and highlights avenues for promoting local economic development across Africa.

## 1. Introduction

In recent years, there has been a notable increase in focus on fiscal decentralization (FD), as researchers and policymakers acknowledge its capacity to stimulate local economic development (LED) in Africa ( Kamara, 2017; Khumalo, 2014); Mosikari and Stungwa (2023). To realize LED, one important approach is for subnational governments (SNGs) in Africa to implement the fundamental principles of FD. These principles include the necessity of transferring authority and autonomy to SNGs to foster decision-making pertaining to the allocation and expenditure of funds within their respective jurisdictions ( Martinez, Qian, Wang, & Zou, 2006). This henceforth stimulates SNGs to generate revenue through varied avenues, including property taxes, user charges, licensing fees, and registration fees. Through the strategic implementation of revenue diversification, SNGs can effectively minimize their reliance on central government transfers, thereby establishing a more resilient and sustainable fiscal framework ( Bello & Mackson, 2023; Martinez-Vazquez, 2008). FD also aids in facilitating the transfer of financial resources from the top tier of government to the regional and local levels. The transfer of funds plays a crucial role in maintaining a balance between the financial demands of various tiers of subnational government ( Bahl, 2000; Martinez-Vazquez & Searle, 2007; Rutto, Minja, & Kosimbei, 2022). It is also important for SNGs to prudently manage their annual expenditures revenues, and transfers to avoid fiscal deficits, thereby posing a significant risk to the financial stability of the country. Faced with fiscal risk, SNGs may receive a bailout from the central government as a means to fulfill their financial obligations whilst continuing their regular operations ( Dabla-Norris, 2006; Oppong, 2020). Under ideal conditions where the core tenets of fiscal decentralization are faithfully executed, even though not a panacea for the developmental challenges confronting numerous African countries at the local level, it still holds the potential to stimulate local economic development ( Haas, Knebelmann, & Nyirakamana, 2021). This potential is particularly significant when harnessed in conjunction with the advantages offered by digital technologies. For example, through the use of digital platforms, governments have been able to streamline administrative processes, improve financial management, and enhance service delivery at the local level. This has resulted in a more efficient allocation of resources, reduced corruption, and increased citizen participation in decision-making processes. Moreover, as technology continues to advance, African countries have the opportunity to further leverage digital innovations to expedite LED and drive economic prosperity at the local level.

The aim of this paper is to explore the prospective role those digital technologies may assume in promoting greater fiscal decentralization and revenue enhancement, particularly via property taxation, to strategically address the challenges of local economic development in Africa. To fulfill this aim, the paper employs a methodical review of the literature to examine the potential of fiscal decentralization and its core pillars to act as driving forces for local economic development within the African context. Through an analysis of best practices and successful case studies from a variety of countries, this paper compiles instances of successful implementation of digital technologies that bolster revenue streams for SNGs while enhancing their governance and institutional capacity.

This paper contributes to the existing body of literature by emphasizing the transformative capabilities of digital technologies in reinforcing fiscal decentralization and property taxation, which in turn catalyzes local economic development in Africa. It further underscores that facilitating economic expansion at the local level can promote Sustainable Development Goals (SDGs) through various avenues. These include generating own sources of revenue via local taxes (e.g., property taxes), service charges, and licensing fees; enhanced delivery of public services; increased local accountability; and the creation of policies designed to attract investments, support local enterprises, or advance specific industries or sectors. Such strategies can give rise to employment opportunities, stimulate entrepreneurship, and boost productivity. These elements contribute to alleviating poverty, promoting social inclusion, and improving living standards, thereby addressing several critical components of the SDGs.

This paper is organized as follows: Section 2 presents a comprehensive overview of the systematic review methodology, as guided by the PRISMA 2020 framework. Section 3 provides an overview of how the core tenets of fiscal decentralization can act as catalysts for local economic development. Section 4 delves into the challenges faced by SNGs that impede local economic development. Section 5 details how digital technology can be strategically employed to promote greater fiscal decentralization and revenue enhancement in response to the local economic development challenges outlined in Section 4. Lastly, Section 6 offers concluding remarks and discusses potential policy implications.

## 2. Methods

This study employed a systematic review methodology, designed to investigate the field of fiscal decentralization in Africa. This investigative process entailed a comprehensive search aimed at delineating the scope of existing scholarship within this field.

For the literature review search the following keywords were used by Monkam and Mangwanya (2024): “fiscal decentralization”, “revenue generation”, “local economic development” and “service delivery” within the African context. Additionally, the search extended to include “digital technologies” and their implications for “subnational government’” in a global framework.

This study involved a thorough analysis of both successful practices and challenges in the field of fiscal decentralization. The identification of these challenges underscores the critical need for SNGs across Africa to embrace digital technologies. Through the utilization of digital tools, SNGs can improve their revenue collection mechanisms, especially focusing on property taxation.

### 2.1 Initial search parameters

The literature review comprehensively integrates a wide array of sources, ranging from peer-reviewed academic journals and conference proceedings to dissertations, policy briefs, and digitally accessible materials. These materials include newspaper articles, blogs, and reports, all hyperlinked within the text for direct reference. This inclusive approach ensures a multidimensional examination of the topic, facilitating a rich and nuanced understanding rooted in scholarly discourse and broader societal reflections. By embracing diverse sources, the review aims to construct a holistic perspective, bridging academic rigor with real-world applications and interpretations.

The primary repository for this search was Google Scholar, covering the period from May 2023 to August 2023. To capture the evolving discourse on fiscal decentralization and provide a comprehensive overview of scholarly perspectives, the temporal filter for publications was set from 2006 to the present.

**Figure 1.**
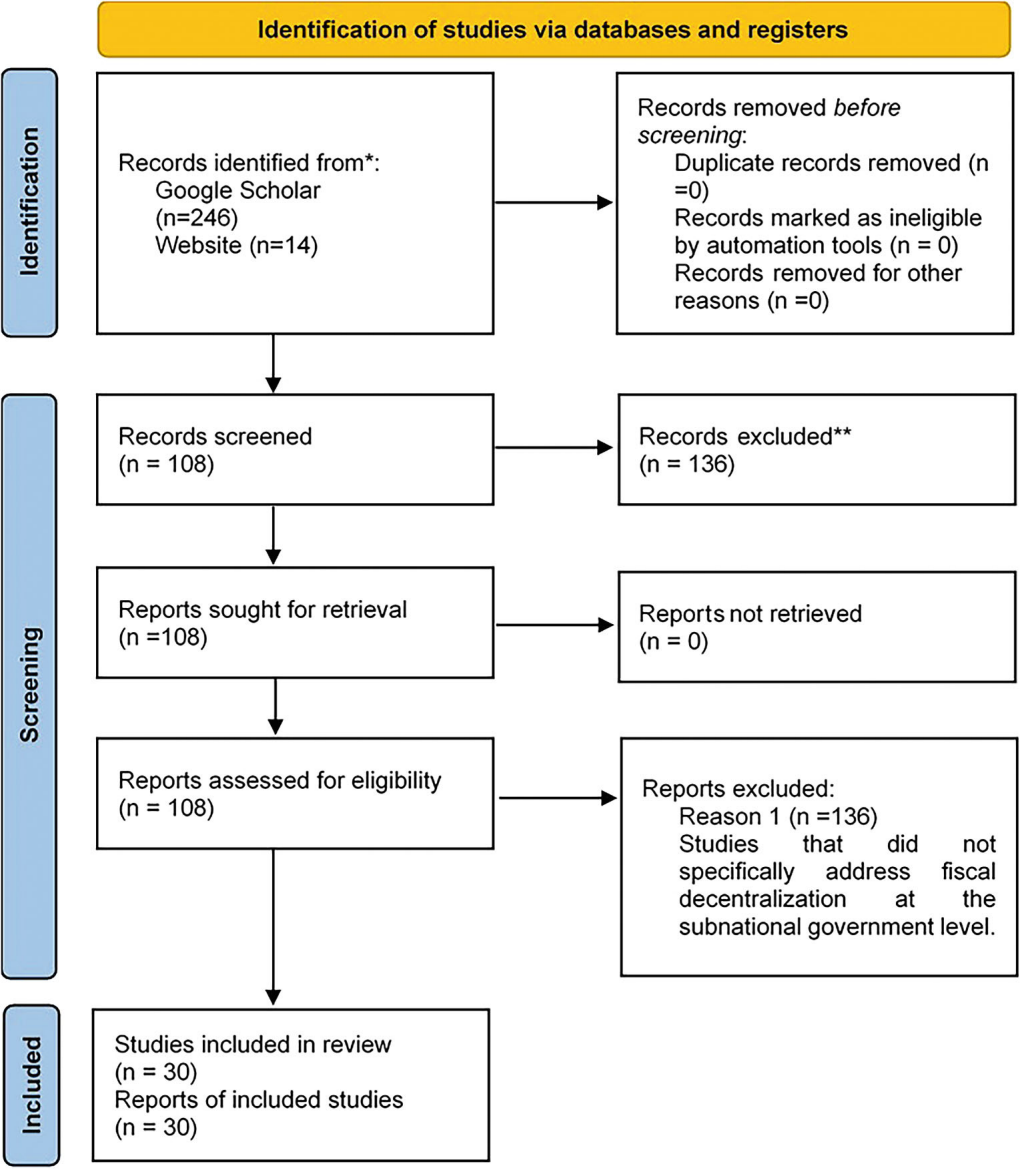
PRISMA framework.

In conducting this research, an initial search yielded 246 sources, with an additional 14 websites considered. After careful examination of abstracts, findings, and conclusions for relevance, 108 sources were identified as pertinent to the study’s focus. This selection process aimed to gather insights into best practices and challenges of fiscal decentralization in Africa, as well as identify successful examples of digital technology adoption. Among these 108 sources, 30 case studies were specifically chosen to highlight these practices, providing concrete examples for analysis. The remaining 138 sources were excluded as they did align with the research objectives. As depicted in Figure 1, the Prisma framework was systematically applied to guide the execution of the literature review. This approach facilitated a structured and transparent methodology for the identification, screening, and inclusion of relevant studies, thereby ensuring the rigor and reproducibility of the review process ( PRISMA, 2020). EndNote software was instrumental in efficiently organizing and referencing these selected sources, facilitating their systematic arrangement and citation.

### 2.2 Inclusion and exclusion criteria

The inclusion criteria were explicitly defined to ensure the relevance and rigor of the literature review. Included were empirical studies and systematic reviews that:

• Examined the best practices and challenges associated with fiscal decentralization in the African context.
• Investigated the global adoption of digital technologies to enhance property tax systems, providing potential solutions to challenges faced by SNGs in Africa

Conversely, the exclusion criteria were applied to

• Studies that did not specifically address fiscal decentralization and subnational government finance in the African context.

This comprehensive review of the literature establishes the study’s basis in current academic work and identifies unexplored areas that this research intends to address. It contributes to the ongoing discourse surrounding fiscal decentralization and the potential of digital technologies to improve property tax revenue collection.

### 2.3 Limitations

The researchers acknowledge the limitations of the current review, notably the emphasis on digital technologies as a solution to fiscal decentralization challenges. This emphasis may inadvertently overlook non-technological factors such as political will, cultural acceptance, and social dynamics, which could equally influence success in implementing fiscal decentralization measures. However, the paper lays the groundwork for future empirical research aimed at assessing the potential impact of digital technologies in promoting greater fiscal decentralization and revenue mobilization. Such research will serve as a strategic response to local challenges in Africa, providing valuable insights into the holistic factors shaping effective fiscal governance.

## 3. Background: understanding the potential of fiscal decentralization to drive local economic development in Africa

In this section, the objective is to demonstrate how the effective application of fiscal decentralization’s key principles can play a pivotal role in fostering a sustainable environment for local economic development in Africa, thereby bolstering robust economies. We will build upon the premise that effective fiscal decentralization, in addition to its four pillars, necessitates political empowerment (political decentralization) and regulatory empowerment (administrative decentralization) as a foundational basis. The four pillars we will discuss include the distribution of expenditure responsibilities, the assignment of revenue sources, the allocation of intergovernmental fiscal transfers or grants, and the regulations surrounding subnational borrowing, budget deficits, and the accrual of subnational debt.

### 3.1 The potential of political and administrative decentralization for local economic development

The establishment of subnational elections as an approach to political decentralization offers the potential of augmenting the effectiveness of governance by empowering voters with increased autonomy ( Bahl, 2000). Voters have more control and decision-making power in shaping the policies and direction of their communities. When SNG officials are appointed, a system of upward accountability is inadvertently put in place, whereby local officials tend to be liable to the individuals responsible for their appointment. On the other hand, the establishment of SNGs through elections fosters a sense of downward accountability, as they become accountable to the local electorate ( Shrestha, Martinez-Vazquez, & Hankla, 2023). A study carried out by Wagana, Iravo, Nzulwa, and Kihoro (2017) found that there is a positive correlation between political decentralization and the effective delivery of public services such as water and sanitation, health, roads and sewer services at the local level in Kenya. Political decentralization in Kenya has paved the way for greater citizen involvement in shaping policies that directly impact their local water and sanitation services. This participatory approach empowered individuals to enhance their quality of life by advocating for more efficient and effective service delivery.

In addition, civil service organizations (CSOs) play a crucial role as independent “watchdogs” continuously monitoring elected local officials’ performance. They function as an intermediary to mitigate potential conflicts of interest that may arise among local authorities due to political affiliations. CSOs have an integral part in advocating for the communities they represent, as they actively promote citizen participation. This enables local authorities to receive valuable feedback on matters relating to service provision. As a result, they help ensure transparency, accountability, and effective governance within the SNGs ( Daughters & Harper, 2007; Devas, 2005).

Administrative decentralization seeks to redistribute power, responsibility, and financial resources for the delivery of public services among various tiers of governance ( Engdaw, 2022; Mollah, 2007). With administrative or regulatory decentralization, SNGs have control over local civil service matters, which include aspects such as salaries, hiring, and termination of employment. Additionally, they govern the local regulatory framework, implying that decisions concerning the nature and method of service delivery should be determined at the local level. As a result, the likelihood of corruption and mismanagement decreases, and public resources are more likely to be used efficiently and effectively ( Bellé & Ongaro, 2014). As for financial management, SNGs enjoy discretion over spending, albeit with monitoring and oversight facilitated by the central government.

### 3.2 Clarifying expenditure responsibilities for local economic development

The subsidiarity principle suggests that public services should be handled by the lowest level of government capable of addressing that matter effectively. However, there are exceptions where public services might be better managed by a higher level of government for reasons of efficiency or economies of scale, interregional spillovers, and redistribution of income. For instance, services like national defense are usually handled by the central government ( Marshall, 2008). Expenditure assignments therefore require the optimal allocation of responsibilities among different tiers of government pertaining to the provision and delivery of public services ^1^, the policy formulation and regulation, financial management, and administrative functions ^2^ of said services. The optimization of expenditure assignments is commonly accomplished through the allocation of exclusive responsibilities across different tiers of government, as opposed to overlapping responsibilities.

Expenditure assignments in local governments in Africa can have significant direct and indirect impacts on local economic development. These assignments allow local governments to effectively deliver public services and make investments in critical areas like infrastructure, business support, and vocational training, tailored to local needs. By leveraging their understanding of the local context, they can deliver services more efficiently, leading to cost savings and better outcomes. For example, to ensure that citizens have access to basic services in Cote d`Ivoire, the central government devolved revenue raising responsibilities. Linked to this fiscal authority is the responsibility to provide basic services such water and sanitation, health, education and roads ( Sanogo, 2019). In addition, Malawi adopted its National Decentralization Policy (NDP) in 1998 which plays a pivotal role in providing a unique classification of functions and responsibilities between the central and local governments. The NDP delegated the responsibilities of service delivery and governance to local governments in Malawi resulting in enhanced improved autonomy and capacity. This also led to greater local decision-making, more efficient allocation of resources, accountability, governance effectiveness, and local empowerment. The implementation of the NDP was in line with the objective of establishing a well-defined and streamlined allocation of duties, thereby fostering improved provision of public services and facilitating LED in Malawi.

### 3.3 Addressing local needs through strategic revenue generation

The correspondence principle implies that if a local government is responsible for providing a particular service, then it should also have the ability to generate or be assigned the revenue necessary to provide that service ( Bahl & Martinez-Vazquez, 2006). For example, if a local government is responsible for maintaining a local park, then it should have the ability to collect taxes or receive funds (the revenue) from the people who use the park or benefit from it. This ensures that the costs of providing the service are covered by the beneficiaries, and that the local government has enough resources to provide the service effectively.

Local municipalities have the ability to generate revenue through various means including property taxes, user fees such as refuse collection, license and registration fees, as well as public utility charges and admissions to recreational facilities ( Bahl, 2008; Bello & Mackson, 2023). These revenue sources enable SNGs to effectively meet their clearly defined expenditure responsibilities. For example, in recent times, Kenya, Nigeria, and South Africa implemented constitutional changes, including modifications to the legal framework governing local governance and finance, that have had an impact on the manner in which cities in these countries generate own source of revenue, giving them more autonomy and control ( Kithatu-Kiwekete, 2018). Greater autonomy and capacity to generate their own revenue sources will enhance SNGs’ autonomy to align spending decisions with local needs, leading to more effective public services and investments that stimulate local economic activity. Revenue assignment also provides a more stable funding base, crucial for long-term planning and investment. SNGs may collect certain taxes, like property taxes or local business taxes more efficiently due to their close proximity to taxpayers ( Rodríguez-Pose & Krøijer, 2009). Furthermore, this fosters increased accountability to how funds are spent, leading to better governance and resource use. Finally, the incentive to promote LED is heightened for SNGs reliant on their own revenues, as this expands their tax base and boosts their income. Additionally, SNGs can potentially implement more equitable tax policies that consider local conditions and ability to pay.

Despite the given authority to generate revenue, SNGs may fail to achieve anticipated outcomes if there is a lack of sufficient enforcement mechanisms to ensure compliance with tax payment obligations ( Sebele-Mpofu, 2020). The effectiveness of revenue collection methods relies heavily on the cooperation of citizens in fulfilling their tax obligations and responsibilities. Without proper enforcement such as penalties on non-compliance, taxpayers may opt to evade their tax obligation, leading to a negative impact on revenue generation for the SNGs ( Bhushan, Samy, & Medu, 2013). In Sierra Leone, for example, research found a noteworthy correlation between increase in enforcement efforts on tax collections by city councils in the region and a marked increase in compliance. The efficiency of these enforcement initiatives was particularly notable when they were directed towards taxpayers with the highest income ( Prichard, Nyirakamana, & Stewart-Wilson, 2022).

While property tax is commonly recognized as a crucial revenue source for local governments, many countries in Africa still do not rely on this form of taxation for generating revenue. In some countries, property taxes may constitute a small portion of local government revenue due to factors like inadequate administrative capacity, lack of up-to-date property valuations, or difficulties in tax collection. For example, property tax is not the primary revenue source for local governments in Cape Verde, instead the local economy thrives on community-based tourism, which fosters employment opportunities and enables them to generate their own revenues through user fees for water and sewage treatment, as well as from tourism-related activities in resort areas ( Bozzato & Pollice, 2022; López-Guzmán, Borges, & Castillo-Canalejo, 2011).

### 3.4 Strengthening revenue generation through intergovernmental transfer systems

The effectiveness of LED hinges on several critical factors, including the capacity of SNGs to generate revenue and their access to intergovernmental transfer systems. SNGs with the capacity to generate revenue can utilize these transfer systems to collect additional revenue ( Smoke, 2015). This financial advantage allows them to invest in infrastructure projects that create employment opportunities, thus stimulating the local economies through increased tax revenue from the employed citizens. Furthermore, SNGs can explore the option of public-private partnerships to develop and manage assets (public buildings and facilities, water supply systems, transportation infrastructure etc.), which generates revenue through shared profits ( Lee, 2013). Intergovernmental fiscal transfers ^3^ contribute to equalizing fiscal capacities (thus reducing regional inequalities), providing funds for specific programs or projects to be carried out by local governments, encouraging policies or reforms, promoting fiscal autonomy, and enhancing public service delivery at the regional or local level. These mechanisms help reduce fiscal disparities and ensure equitable access to public services across different levels of government.

In South Africa, the Intergovernmental Fiscal Relations Act (1997) assumes a crucial role in the distribution of revenues among the national, provincial, and local governments ( de Visser & de Visser, 2022). This legislation guarantees every municipality’s constitutional entitlement to receive a fair and proportionate allocation of revenue. Municipalities are better able to provide essential services to the community such as healthcare, education, infrastructure development, and social welfare programmes when they receive an equitable share of the revenue. Bvuma and Joseph (2019) noted that the effective provision of essential services by municipalities plays an integral part in fostering the holistic welfare and advancement of the local economy. Similarly, in 2019, Zimbabwe introduced intergovernmental transfers, with Mashonaland Central Province, for instance, receiving 5% of the national revenue ( Munyede, Chikwawawa, & Mazambani, 2021). The province used the funds from the intergovernmental transfers to improve service delivery in education, water, sanitation and health. As part of this initiative, the province undertook key projects such as drilling of boreholes, building classroom blocks, and setting up a new clinic.

### 3.5 Local government borrowing and debt in driving local economic development

If SNGs do not carefully balance their annual expenditures with revenues and transfers, this may result in subnational deficits and the incurrence of subnational debt. SNGs may take on debt through issuing bonds or taking loans, as a way of financing their activities. This can have both direct and indirect impacts on LED in Africa ( Dabla-Norris, 2006). In Ghana, borrowing practices within the Metropolitan, Municipal, and District Assemblies (MMDAs) are bound by some constraints. MMDAs have the ability to secure loans and overdrafts, subject to a prescribed maximum threshold of 2,000 Cedi, without requiring authorization. Any level of borrowing surpassing this specified threshold is subject to stringent limitations and regulatory supervision. In Guinea Bissau, municipalities can engage in borrowing activities through loans and securities (municipal bonds and notes) at all maturities. In Ivory Coast, municipalities are allowed to borrow, although under strict conditions unspecified by the government. In Senegal, the local government has the authority to issue domestic and international bonds and borrow funds contingent on the approval of the central government and a comprehensive evaluation of their financial capabilities.

Borrowing, when used prudently, enables local governments to invest in large-scale infrastructure projects crucial to economic development that may not be feasible with current revenues. The influx of borrowed funds can stimulate the local economy, supporting businesses and creating jobs. Spreading the cost of substantial investments over time via borrowing ensures local governments maintain fiscal sustainability to continue providing services and fostering development ( Smoke, 2019). Furthermore, demonstrating responsible borrowing and debt management can enhance a local government’s creditworthiness, leading to lower borrowing costs and increased access to future financing. Borrowing publicly, such as through issuing bonds, can heighten government accountability as bondholders have a vested interest in the local government’s financial health. Lastly, the obligation to service debt can instill financial discipline, encouraging local governments to manage their finances responsibly and efficiently ( Jose, 2022).

In conclusion, under ideal conditions where the core tenets of fiscal decentralization have been effectively implemented, it holds the potential to stimulate local economic development. However, SNGs in Africa have not yet fully capitalized on this opportunity. They continue to grapple with a myriad of obstacles tied to fiscal decentralization that hinder the effective realization of local economic development. It is nonetheless important to underscore that the impacts of fiscal decentralization can depend on many factors, including the extent and quality of the decentralization process, the capacity of local governments, and the broader political and economic context. It is also possible for decentralization to have negative impacts, for example, if it leads to a duplication of efforts, increased corruption, or greater regional inequalities. The next section explores the challenges SNGs face in achieving local economic development in Africa.

## 4. Fiscal decentralization challenges impeding local economic development in Africa

In the African context, numerous challenges can impede the successful application of fiscal decentralization principles and their ensuing positive impacts on local economic development. This section highlights the key obstacles tied to fiscal decentralization that local governments across Africa grapple with, obstructing the effective implementation of local economic development initiatives.

### 4.1 Governance and institutional capacity

Many local governments in Africa face challenges due to weak institutional capacity, which often results in inefficiencies, poor service delivery, and missed local economic development opportunities due to lack of administrative and technical capacity. Additionally, corruption and governance issues present significant problems in some areas, siphoning resources away from developmental efforts and eroding public trust in institutions.

For example, according to Thusi and Selepe (2023), the failure of effective governance in South Africa can be attributed to shortcomings in knowledge and skills, corruption, the practice of cadre deployment ^4^, and the erratic nature of coalitions ^5^ at the local government level.

According to South Africa’s Auditor General “the local government has been characterized by dysfunctional municipalities, financial mismanagement, administrative instability, and crumbling municipal infrastructure. This leads to deteriorating standards of living and service delivery failures, resulting in protests”. The effectiveness and productivity of public services have been hindered by various challenges, including the late registration of projects, irregularities in procurement practices [which undermine the fairness and transparency of the process], and council decisions. This has posed significant challenges to the overall functioning of public services. In addition, municipalities are experiencing financial losses as a result of inefficient revenue billing and collection processes, inequitable procurement practices, and expenditures made for goods and services that were either unused or not received.

This South African experience highlights other local governance issues that significantly impact municipal finance, service delivery, and overall development in Africa. First, many local governments grapple with transparency and accountability issues, often due to inaccessible information about government operations and budgets. Coupled with inadequate citizen participation in decision-making processes, this can foster corruption and impede accountability ( Managa, 2012). Second, many local governments lack the institutional capacity for effective governance (including well-trained staff, robust administrative systems, and sound financial management practices), leading to inefficiencies and poor service delivery. Third, the legal and regulatory frameworks that govern local governments can sometimes be inadequate or inconsistently applied, causing uncertainty and disputes ( Masuku & Jili, 2019). Finally, unclear roles and responsibilities in inter-governmental relations can also hinder the autonomy local governments need to respond effectively to local needs ( Atkinson, 2021).

Research institutions could play a crucial role in strengthening effective governance by offering cutting-edge analysis and recommendations to increase decision-making processes and operational effectiveness ( Nkrumah, 2022). According to Vilakazi and Adetiba (2020), there is not enough collaboration between research institutions and the government in South Africa, resulting in some policies becoming incompatible with local government’s objectives. To address this issue, researchers can conduct comprehensive studies that assist policy makers in developing evidence-based strategies, enabling the successful implementation and execution of policies that genuinely meet the needs of the communities they serve.

### 4.2 Revenue generation and enforcement

Many local governments in Africa experience limited revenue generation due to factors like a small tax base, widespread informality, poor tax compliance, and weak tax administration, resulting in heavy dependence on often unreliable central government transfers ( Awasthi, Nagarajan, & Deininger, 2021). Moreover, these fiscal transfers often fall short of what is needed to fulfill devolved responsibilities, leading to a financial strain on local governments. Despite legislated fiscal decentralization processes, local governments frequently lack tax autonomy as central governments tend to retain control over local tax policy and administration, such as setting tax rates ( Masaki, 2018). With financial autonomy, SNGs are entrusted with the authority to exercise independent judgment pertaining to their revenue, expenditures, investments, and financial targets. Consequently, this diminishes their dependence on external entities for financial support. For example, the majority of teachers in Tanzania, according to a study conducted by Mtasigazya (2019), contended that the council lacked autonomy in financial management and authority over funds allocated for primary education service delivery. Furthermore, a study by Lameck and Kinemo (2021) argued that local governments in Tanzania struggled to generate sufficient revenue, making them reliant on intergovernmental transfers. Yet, even with this supplemental funding, to the resources fell short of meeting the growing demands for urban services and infrastructure.

SNGs often attempt to foster tax compliance by strategically prioritizing immovable property taxation, recognizing its potential as a lucrative revenue generator ( Collin, Di Maro, Evans, & Manang, 2022). However, people are often reluctant to pay property taxes because they do not see any tangible benefits, particularly in areas characterized by poor infrastructure with sporadic or non-existent service delivery ( Monkam & Moore, 2015). Madagascar’s efforts to mobilize property tax revenue are severely constrained by limitations on the operational capacities of local governments in various facets of property taxation, including property identification, valuation, collection, enforcement mechanisms, and dispute resolution. Some studies in Tanzania and Kenya, for example, Collin et al. (2022) and Mwangi (2019), found that property tax policies were often neglected due to taxpayers’ skepticism about the government’s use of the revenue. With a focus on larger properties, local authorities in both countries had a limited tax base, resulting in reduced revenue and compromised service delivery efficiency.

While revenue collection can be influenced by political, economic, and technical factors, it is crucial to recognize the significant impact of the COVID-19 pandemic on both local and global economic activities. The financial strain from the pandemic reduced individuals’ ability to meet tax obligations, further impeding tax compliance. For instance, in South Africa, the extended lockdown is estimated to have decreased tax revenue by ZAR285 billion ( Madhi et al., 2020). This significantly impacted local governments by reducing intergovernmental transfers, leading to a shortfall in the effective delivery of services. Furthermore, poor compliance with tax obligations, coupled with limited enforcement and the administrative challenges of local governments, has hindered regional economic growth. When local governments are unable to harness adequate revenue, it poses a major obstacle their agility in responding to emerging needs, their capacity to allocate resources toward critical sectors and nurture domestic enterprises. This is not merely a fiscal challenge; it is a bottleneck to holistic regional development, stymieing innovation, and impeding the realization of broader economic potential ( Martínez-Córdoba, Benito, & García-Sánchez, 2021).

Other local economic development challenges include rapid urbanization, which strains local governments’ capacity to deliver essential services, manage urban planning, and promote sustainable development. Additionally, high levels of poverty and inequality can significantly impede local economic development, as they can restrict the local tax base, increase the demand for public services, and lead to socio-economic exclusion. Moreover, environmental challenges, including climate change, pollution, and natural resource depletion, pose significant hurdles for local economic development, with local governments often lacking the resources and capacity to tackle these issues effectively.

### 4.3 Political and administrative empowerment

In the context of Africa, several challenges can hinder the successful implementation of these forms of decentralization and their positive effects on local economic development. Numerous local governments in Africa face capacity constraints, lacking the human, financial, and technical resources necessary to fully assume the responsibilities that come with decentralization. This can lead to inefficiencies, poor service delivery, and challenges in implementing local development projects. Furthermore, political interference from higher government levels can undermine decentralization, limiting local governments’ autonomy and distorting local development priorities ( Abdulai, Tundyiridam, & Alhassan, 2016; Otu & Anam, 2019; Phillip & Ajibade, 2016). In the Katima Mulilo Town Council (KMTC) of Namibia, local officials were appointed based on political affiliations. This politicization compromised the efficiency and effectiveness of service delivery, stemming for example, from a lack of foresight in knowledge management ( Kalonda & Govender, 2021). It is frequently observed that leaders tend to give higher priority to political interests as opposed to the implementation of efficient management principles, resulting in negative impact on the provision of public services ( Ndevu & Muller, 2018). advanced that political instability has an adverse effect on civic tax compliance, thereby eroding the credibility of government institutions, particularly those mandated with tax administration. The author substantiated this argument by providing instances of civil conflicts in Burundi and Rwanda, which led to a reduction in tax-generated revenue.

The Mogalakwena municipality situated in the Limpopo Province of South Africa also demonstrated that political conflicts, initially perceived as confined to the national level, permeate subnational governments. Consequently, officials operating within these SNGs find themselves immersed in an environment characterized by intense politicization, polarization, and poor working conditions. This inevitably creates an impact on the capacity of SNGs to provide public goods and services ( Chilenga-Butao, 2020). In Zimbabwe, the selection of councilors responsible for the administration of local authorities primarily hinges on their political party affiliations, often resulting in a lack of the requisite technical proficiency essential for sound policy formulation and effective execution. Although there are no specific qualifications required, these councilors are responsible for supervising the activities of local authorities. As a result, the management of local authorities in Zimbabwe lacks professionalism and instead is often subject to political biases, driven by politicians’ concerns over potential political protests.

When elected, local authorities must ensure the effectiveness and productivity of service delivery to be accountable to their constituents. However, in certain instances, the challenge arises when attempting to terminate or impose disciplinary measures on the individuals. In their study in Namibia, Kalonda and Govender (2021) also highlighted that a significant proportion of the participants in their study noted that KMTC faced administrative challenges and municipal authorities were aware of these issues, though failed to take appropriate action due to a lack of control and insufficient collaboration with the community.

### 4.4 Civil service skills development

As underscored in prior sections, weak institutional capacity is a prevalent challenge among numerous subnational governments in Africa. This is particularly apparent in their insufficient human capacity to fully embrace the duties associated with fiscal decentralization and to manage their finances and affairs effectively ( Kwabena Obeng, 2021).

The lack of skilled human capital in South African municipalities, for example, has caused problems with the provision of services, thus limiting the availability of vital amenities across various communities ( Masuku & Jili, 2019). There is an apparent shortage of skilled personnel including civil engineers, technologists, ^6^ and technicians, which affects the maintenance and improvement of infrastructure within local municipalities. The shortage of capacity is notably apparent in key sectors such as water and sewage treatment facilities, distribution networks, and transportation infrastructure. For example, limited resources and a shortage of expertise have led to a cholera outbreak in the Hammanskraal region, situated in the Northern Gauteng Province of South Africa. This was a result of the fact that not enough planning, maintenance, and updates were done on the existing facilities. To address these challenges, information technology (IT) can offer solutions. For example, SALGA is using a digital factory, supported by cloud-based technology, to develop and implement innovative solutions that address specific challenges. This initiative, which has been gaining traction, is part of SALGA’s strategy to enhance service delivery to South Africa’s citizens. In addition, IT can play a vital role in identifying high-risk cholera prone areas through data analytics and predictive analytics ( Boukenze, Mousannif, & Haqiq, 2016). Health professionals can leverage mobile applications and popular online platforms such as Twitter (now referred to as “X”), Facebook, and TikTok to disseminate essential information on cholera prevention and management. By tapping into technology and the reach of social media, we can enhance our efforts to contain cholera and safeguard public health. Despite the potential solutions offered by IT, progress remains hindered by the shortage of skilled professionals capable of utilizing these solutions within local governance. The slow adoption of efficient digital solutions for enhancing service delivery continues to stall progress.

Regarding property taxation, the City of Harare in Zimbabwe, for example, has experienced challenges in retaining skilled personnel, particularly engineers and valuators. As a result, the city has become dependent on external valuators to assess property values ( Nengeze, 2018). In addition, local government authorities’ struggles with revenue collection are not just a matter of capability but also of commitment. As highlighted by Nkuna (2021), the root of this challenge lies in the inadequate incentives and low salaries. When revenue collection falters due to lack of motivation, this does not only limit infrastructure development and the provision of essential services but also erodes public trust.

A study conducted by Thusi and Selepe (2023) revealed various disconcerting aspects related to appointment practices, skill development programmes, and institutional management across South African municipalities. The study highlighted a prevalence of irregular and/or unsuitable appointments, coupled with below-average competencies and insufficient initiatives to foster skill development. Moreover, the research revealed that while 7% of municipalities opted for the utilization of consultants to address staffing shortages during the 2020-21 period, 62% of municipalities enlisted the services of consultants to mitigate the deficiency in expertise within their finance departments. The utilization of consultants in the remaining 31% of municipalities was attributed to a scarcity of skills and a vacancy gap.

Furthermore, staff changes, including retirements, transfers, and resignations, have eroded the institutional memory of local governments. As seasoned professionals depart, they take with them a wealth of expertise, leaving a knowledge void that could have been pivotal in addressing the multifaceted challenges faced by SNGs ( Cong & Pandya, 2003; Obwaka, Kwanya, & Mwai, 2019). Take South Africa’s local government as a case in point; it is not just grappling with the immediate loss of knowledge from retirements but also with the longer-term implications of not having systems in place to capture and leverage this invaluable intellectual capital ( Mothamaha & Govender, 2014). Moreover, in both South Africa and Zimbabwe, local governments seem hesitant to share knowledge among employees. This reluctance is rooted in a pervasive mistrust among employees and stakeholders within these authorities, raising critical questions about the foundational trust and transparency within these governmental structures ( Mothamaha & Govender, 2014; Mutandwa & Hendriks, 2022).

Having outlined the major obstacles that can obstruct the successful execution of fiscal decentralization and its beneficial impacts on local economic development, the subsequent section presents potential strategies to counteract these challenges.

## 5. Leveraging digital technology for fiscal decentralization and property taxation in Africa

In this section, we delve into the prospective role those digital technologies may assume in promoting greater fiscal decentralization and revenue enhancement, specially through property taxation, as a strategic response to the local economic development challenges in Africa.

While certain African nations grapple with technological advancements, they possess the potential to adopt cost-effective technologies that align with their limited resources. Through strategic and judicious adoption of these technologies, these countries have the potential to narrow the digital divide and harness technology to tackle their unique challenges tied to fiscal decentralization and property taxation. The introduction of technology has been linked to enhanced administration of land and as in low- and middle-income countries, demonstrating that they can readily adapt to technological changes ( Bahl, McCluskey, & Franzsen, 2022). Despite the slower pace of technological progress in some African nations others have embraced digital technologies to strengthen fiscal decentralization and implement property tax reforms that promote local economic development ( Joubert, Murawski, & Bick, 2023). These countries have made substantial investments towards creating robust digital infrastructure, successfully employing digital platforms for property tax administration. Furthermore, they have harnessed mobile technology to streamline and accelerate property tax administration processes.

Developing countries possess untapped revenue potential through property taxation, yet the realization of this potential often meets significant challenges, such as prolonged and cumbersome administrative procedures and unfavorable political incentives such as political interference and lack of political will, which can delay substantial property tax revenue accumulation ( Kangave, Occhiali, & Kamara, 2023). However, the introduction of technological innovations in property registration, valuation, and tax compliance has simplified these procedures, reducing associated paperwork and administrative burdens.

So, while digital platforms can streamline property registration, advanced algorithms and data analytics have the capacity to improve the accuracy of property identification and valuations, considering factors such as geographical location and current market trends. Automated systems facilitate monitoring of property tax obligations and identifying non-compliance by tracking transactions and changes in ownership. These technological advancements hold the potential to boost operational efficiency, enhance transparency, and encourage compliance with tax regulations, thereby streamlining and optimizing property tax collection procedures ( Saragih, Reyhani, Setyowati, & Hendrawan, 2022). Governments worldwide are increasingly integrating Artificial Intelligence (AI) and machine learning into their operations, thereby facilitating significant enhancements in public services, including property taxation. For instance, tax authorities are employing AI-powered systems to autonomously analyze extensive volumes of financial data and identify potential tax evasion or fraudulent activities. Sophisticated algorithms facilitate rapid detection of patterns and anomalies, enabling governments to promptly undertake necessary measures and uphold adherence to tax regulations ( Henman, 2020).

Furthermore, AI-driven chatbots and virtual assistants are being leveraged to assist taxpayers in navigating complex tax codes, providing tailored guidance, and addressing inquiries. This improvement in user experience not only enhances overall satisfaction but also reduces the workload on government call centers. The integration of AI and machine learning (ML) into property taxation systems empowers governments to bolster their revenue generation, enhance compliance levels, and provide citizens with more streamlined and impactful services ( Engstrom, Ho, Sharkey, & Cuéllar, 2020; Henman, 2020).

### 5.1 Strengthening institutional capacity through digital innovation

Digital technologies, artificial intelligence (AI), and machine learning (ML) hold transformative potential for strengthening the institutional capacity of SNGs in Africa. Institutional capacity is key to the effective functioning of local governments, encompassing areas such as administrative efficiency, financial management, human resource development, and data management.

By automating routine tasks and improving the accuracy of administrative functions, digital technologies can significantly enhance the efficiency and productivity of local government employees. AI-powered software, for instance, can automate functions like data entry, document processing, and financial reporting, thereby increasing the efficiency of local government operations and allowing staff to focus on strategic tasks. In a study by Yigitcanlar, Agdas, and Degirmenci (2023), city managers in Australia and United States of America expressed optimism regarding the potential of AI to deliver improved efficiency within local governance. For example, in the local government context, AI is being utilized in various ways, such as managing city assets through structural health monitoring, detecting and diagnosing energy infrastructure faults, enhancing customer service accessibility with chatbots, and automating transportation with autonomous shuttle buses ( Yigitcanlar, Wilson, & Kamruzzaman, 2019). The overall process can be bolstered through e-government, i.e., the deliberate use of ICT within the sphere of public administration and political decision-making processes ( Von Haldenwang, 2004).

In addition, digital platforms can be instrumental in building the human capacity of local governments by providing online training and skills development. Such platforms can enable staff to acquire essential skills in areas like strategic planning, financial management, project management, and data analysis, thereby augmenting their capacity to serve their communities effectively ( Sivarajah, Irani, & Weerakkody, 2015). By analyzing large volumes of data, digital technologies, through predictive analytics, can forecast revenue, expenditure, and demand for public services, thereby generating insights that inform policy decisions, and assist with budgeting and planning. Additionally, digital technologies can streamline financial management processes. For example, blockchain technology can increase transparency and reduce fraud in financial transactions, which is critical for the prudent management of public resources. It can also offer meticulous tracking, documentation, and security for each stage of the procurement process on an immutable platform. By implementing blockchain, an unalterable “digital ledger” can be created. This ledger serves as a powerful tool for public sector auditors, providing an unimpeachable record of transactions and contributing to increased transparency and accountability ( Kahn, Baron, & Vieyra, 2018). Furthermore, digital technologies can increase transparency and accountability by making government data open and accessible. Open data portals, for instance, can provide citizens with information on local government budgets, expenditures, and performance metrics.

AI and ML can also optimize the delivery of public services. For instance, geospatial data can help identify areas in greatest need of services such as healthcare, education, and infrastructure, allowing for more efficient and effective allocation of resources ( Anshari & Hamdan, 2022). Lastly, digital platforms can enhance citizen engagement by providing channels for feedback and participation in local decision-making. For example, mobile apps can enable citizens to report service delivery issues, pay taxes, and access information about local government services. In Uruguay, a mobile and web-based platform application called “Por Mi Barrio” was launched in 2015 to allow residents of Montevideo to report issues, such as public infrastructure malfunctions or acts of vandalism. These reported issues, along with the government’s corresponding responses, are visually represented on a publicly accessible website in the form of a map. This allows for public visibility and accountability and enables more direct and effective community involvement in local governance ( Peixoto & Fox, 2016). In addition, e-Government tools can be leveraged to emphasize transparency and participation as central principles guiding municipal-citizen interactions. Portugal’s high e-Government development index, for example, reflects the public sector’s outstanding performance in efficiency, effectiveness, and service delivery, a success significantly tied to the adoption of e-Government strategies ( Tejedo-Romero et al., 2022).

South Africa has taken proactive steps to bridge the skills gap within its public sector through the establishment of the National School of Government. This institution provides robust training and development programs designed for public servants at all tiers - national, provincial, and local ( Msomi, Munapo, & Choga, 2016). To enhance the reach and flexibility of these educational programs, the school has integrated synchronous e-learning methodologies. ^7^ Notably, such e-learning platforms are already having a positive impact in countries like the USA, Canada, and Korea, where civil servants have harnessed these digital platforms to boost their competencies and enhance the efficiency of service delivery ( Lai, 2017).

Big data, according to Androutsopoulou and Charalabidis (2018) presents a significant opportunity to address the skills gap particularly in the development of policies that effectively cater to constituent needs. The authors propose a methodological framework for leveraging big data in policymaking, consisting of seven distinct stages: problem identification, data understanding, data mining, policy development and modeling, policy simulation, results visualization, and knowledge consolidation. This approach underscores the potential of evidence-based policies to deliver the intended outcomes. Collaborations between governments and data scientists or analysts could be pivotal. Experts in data science can examine large datasets to inform effective policy decisions, using statistical analysis and machine learning techniques. A case in point is Brazil’s Observatory of Public Spending, under the Office of the Comptroller General. This body successfully integrated data mining tools, enabling government officials to audit a vast amount of public expenditure, approximately US$5 trillion. In 2015, this process unearthed over 7,500 cases of unclean audits which amounted to US$104 million ( Kahn et al., 2018). The example highlights the transformative potential of big data in enhancing governance and improving public spending efficiency ( Tejedo-Romero, Araujo, Tejada, & Ramírez, 2022).

### 5.2 Maximizing revenue through digital transformation

The advent of digital technologies has ushered in transformative opportunities for local governments in Africa to enhance their revenue base, improve enhance their tax systems’ efficiency and fairness, and drive local economic development. The application of these technologies, when done strategically, can help to rectify inefficiencies and loopholes within the existing fiscal systems.

First, the ability to expand the local tax base is a significant advantage provided by digital technology. By harnessing and analyzing data from various sources, potential taxpayers who are currently not in the system can be identified. Here, machine learning algorithms can play a crucial role by automating the scrutiny of large datasets to pinpoint discrepancies, thereby uncovering businesses and individuals who may be under-reporting or evading taxes ( Politou, Alepis, & Patsakis, 2019). Second, improvements in tax administration can be achieved through the automation and streamlining of processes, reducing bureaucratic inefficiencies and human error ( Tetteh, 2012; Xusan o‘g‘li, 2023). Software solutions can manage taxpayer databases, calculate tax liabilities, generate tax bills, and track payments. The integration of AI can further enhance these processes by predicting future revenue trends and identifying potential issues before they escalate.

In the context of property identification and assessment, digital technologies can be transformative. One of the main challenges in property taxation is accurately identifying properties and estimating their value. In many African cities, property records are incomplete or out of date, and valuation methods are often rudimentary. Geographical Information Systems (GIS) and remote sensing technologies can be used to create detailed maps of properties, ensuring the accuracy and fairness of property tax assessments ( Lambin et al., 2014). For example, spatial data (information about the physical location and shape of geographic features) and remote sensing data (information collected from satellites or airborne sensors) from datasets such as Copernicus and World Settlement Footprint on land use and settlement patterns, can be used to identify and assess properties and their characteristics for tax purposes ( Csorba, Bánóczki, & Túri, 2022; Marconcini, Metz-Marconcini, Esch, & Gorelick, 2021). Night-Time Light Data can be used to assess the level of development and economic activity in a given area, which can inform property valuations ( Keola, Andersson, & Hall, 2015). Google Open Building and AID data offer detailed building and infrastructure data that can also be used in property assessments. Geo-boundaries and GADM data could provide detailed geographic boundaries, which can help local governments analyze revenue data at different geographic levels, such as neighborhoods or districts. This can support more granular and targeted policymaking. By combining this data with AI and ML techniques, local governments can automate and identify properties currently outside the tax system, thus expanding the property tax base. They determine appropriate tax values, considering factors such as property size, location, and market trends. This will lead to more equitable and efficient property taxation and increased revenues.

Digital enhancements in tax collection can be manifested in the form of e-payment systems, making it easier for taxpayers to fulfill their obligations and increasing compliance rates ( McCluskey, Franzsen, Kabinga, & Kasese, 2018). AI can also be used to identify late-paying taxpayers, allowing for targeted and timely enforcement actions. These enforcement mechanisms can also benefit from AI and ML by identifying patterns of non-compliance and predicting potential defaults ( Kamil, 2022).

Finally, digital platforms can significantly improve services for taxpayers, providing easy access to information, facilitating tax filings and payments, and enabling communication with tax authorities. AI-driven chatbots can offer round-the-clock customer support, answering common queries and guiding taxpayers through complex processes. For example, Digital Financial Services (DFS) have seen significant adoption in Africa, bringing a host of economic benefits. One major advantage, as highlighted by Santoro, Amine, and Magongo (2022), is the ability to monitor transactions through digital pathways, such as mobile money. With the integration of third-party data, DFS can improve the efficiency of verifying property tax declarations and payments. This can pave the way for data-driven audits and promote transparent property tax administration. Moreover, the mandatory implementation of e-filing for tax compliance in some countries, combined with DFS, can enhance financial administration by improving operational effectiveness, transparency, compliance, and inclusivity. As a result, resource allocation becomes more efficient, decision-making improves, and sustainable economic growth is encouraged ( Munoz, Mascagni, Prichard, & Santoro, 2022). The following are examples of countries that have effectively leveraged digital technologies to enhance local revenue generation.

In Rwanda, drones have been utilized for collecting land data, leading to significant improvements in property surveying and registration. These unmanned aerial vehicles (UAVs) enable the acquisition of highly accurate images, facilitating the identification and demarcation of property boundaries. Such advancements have positively impacted sectors like construction and agriculture, as well as land information systems ( Mihgo & Magina, 2022). Similarly, Zanzibar implemented a comprehensive fiscal cadastre using drone imagery between 2011 and 2016, fostering transparency and mitigating the risk of property data manipulation and mismanagement.

In Warsaw, Poland, local government officials have utilized GIS and remote sensing technology to collect critical data regarding building characteristics such as the number of storeys and the technical state of facades, which has significantly improved the accuracy of property tax assessments ( Dąbrowski & Latos, 2015). Similarly, Sierra Leone, Malawi, and Lagos State in Nigeria have incorporated IT systems into their administrative processes. These systems monitor data input, identifying any discrepancies in valuation or payment data, and flag potential misconduct. As a result, these countries have been able to enhance their property tax administration processes, including enforcement strategies, and boost revenue collection.

In countries like Kenya and Zambia, local government authorities (LGAs) have autonomy to develop indigenous ICT solutions or adopt Commercial Off-The-Shelf (COTS) solutions ( McCluskey et al., 2018). This autonomy empowers them to devise innovative and cost-effective approaches to use technology to improve governance and service delivery. For instance, Kenya has successfully developed CountyPro, a software solution designed to optimize county revenue management efficiently. This web-based e-governance solution facilitates smooth information exchange among various county departments, marking a shift towards effective governance. It provides a user-friendly online interface for citizens and a specialized administrative interface for county personnel ( McCluskey et al., 2018).

### 5.3 Digital technologies and public service delivery

Digital technologies, along with AI and machine learning, also offer transformative potential for enhancing public service delivery in local governments across Africa. Leveraging these technologies with spatial data, such as the geocoded Afrobarometer data, may equip local governments with insights into their service landscapes, helps identify gaps, and provides strategic direction for more effective and efficient service planning and delivery ( Konte & Vincent, 2021).

The first significant utility of spatial data is in identifying service gaps. Data from sources such as Copernicus, World Settlement Footprint, or Google Open Building can provide exhaustive information about the location of residential and commercial establishments. When integrated with AID data, Geo-boundaries, and GADM data, which detail the location of public services like clinics, hospitals, and police stations, by overlaying these data sets, local governments can identify areas that are underserved and need additional services. Secondly, understanding proximity of essential services to communities is a key factor in accessibility. Machine learning algorithms can analyze spatial data to calculate the distance from each household or business to the nearest service point. This assists local governments in identifying areas where services are not easily accessible, and subsequently, where service accessibility needs to be improved ( Müller et al., 2018; Sumanta, 2022).

The planning of new services also substantially benefits from spatial data. By identifying areas experiencing rapid growth but with limited access to essential services, local governments can strategically plan and locate new service centers, such as health clinics, to cater to this expanding population. Efficiency in service delivery is another critical area where AI and ML can make a significant difference. These technologies can optimize routes for service delivery vehicles, such as garbage trucks or ambulances, based on real-time traffic data and the location of households or incidents, leading to improved operational efficiency ( Akanbi & Agunbiade, 2013).

Understanding public sentiment is crucial for any government, and geocoded Afrobarometer data can provide invaluable insights into citizen perceptions of public services. This feedback can help local governments prioritize improvements in areas where citizens express dissatisfaction or concerns ( Konte & Vincent, 2021). Lastly, AI and ML can be employed to predict future demand for public services by analyzing trends and patterns in spatial and Afrobarometer data. This foresight allows local governments to plan resources and infrastructure effectively to meet anticipated demand ( Burke, Driscoll, Lobell, & Ermon, 2021).

In conclusion, the strategic application of digital technologies presents a significant opportunity for local governments in Africa to bolster fiscal decentralization and revenue enhancement, particularly through property taxation. These tools can be leveraged to address local economic development challenges, ultimately leading to more sustainable and inclusive growth. However, the successful deployment of these technologies’ hinges on several essential factors. Firstly, the provision of robust digital infrastructure is vital to support the wide-scale implementation and optimal utilization of these technologies. Secondly, it is crucial to build a workforce that possesses the skills and knowledge required to leverage these digital tools effectively. This requires substantial investment in education and training programs, as well as a commitment to ongoing professional development. In addition, a conducive legal and regulatory environment is necessary to foster the growth and application of digital technologies ( Sousa & Rocha, 2019). Policymakers must ensure that regulations are adaptable and responsive to the rapidly changing digital landscape, while also safeguarding citizens’ rights and interests. Data privacy and security are paramount, particularly given the sensitive nature of the data involved. As such, robust measures should be put in place to protect data and prevent breaches, which can undermine public trust and impede the uptake of digital services ( Romansky, 2021).

Finally, it is important to ensure digital inclusivity, so that all citizens, including those who are marginalized or digitally excluded, can benefit from the improvements in service delivery that these technologies enable. This may involve initiatives to improve digital literacy and access among these groups. Despite the significant potential that digital technologies, AI, and ML offer, these challenges must be acknowledged and addressed to fully harness their benefits. With thoughtful planning, investment, and policy frameworks that support their effective implementation, these tools can make a meaningful contribution to enhancing fiscal decentralization, boosting revenue, and promoting local economic development in Africa ( Latif, Qadir, Farooq, & Imran, 2017).

## 6. Conclusion and policy recommendations

This paper delves into the transformative potential of fiscal decentralization as a catalyst for local economic development across Africa, a subject of growing importance and multifaceted complexity. Through a methodical review of existing literature, the study explores both the challenges and opportunities inherent in fiscal decentralization, with a particular emphasis on the pivotal role of digital technologies. By enhancing our understanding and application of these concepts within the African context, the study concludes that investment in digital infrastructure, skills, and robust regulatory frameworks, coupled with judicious consideration of data privacy and security concerns, can empower subnational governments in Africa. This enables them to harness the power of fiscal decentralization to foster sustainable growth and development.

This paper contributes a valuable perspective to the existing literature, offering actionable insights and policy recommendations that can guide policymakers, scholars, and practitioners in their concerted efforts to realize the full potential of fiscal decentralization in Africa. Firstly, to successfully implement fiscal decentralization and enhance its impacts on local economic development in Africa, it is crucial that local governments build robust institutional capacities. This involves the provision of training and development programs aimed at enhancing skills in fiscal management, tax administration, and governance. Additionally, the establishment of efficient administrative systems is key to ensuring smooth operations. As part of this capacity-building effort, governments must also invest in the development of reliable digital infrastructure, including internet connectivity, data centers, and the necessary hardware for digital technologies, artificial intelligence (AI), and machine learning (ML) applications.

Secondly, governments should also focus on creating supportive legal and regulatory frameworks. These would clearly define the fiscal responsibilities and rights of local governments and encourage transparency and accountability in their operations. Additionally, leveraging digital technologies, AI, and ML can greatly enhance revenue generation, particularly in the area of property taxation. By improving the accuracy of tax assessments, streamlining tax collection processes, and reducing tax evasion, these technologies can play a pivotal role in revenue enhancement. However, as digital technologies become more integrated into fiscal management and public service delivery, stringent measures to ensure data privacy and security, including the use of secure databases, encryption, and regular cybersecurity audits, must be in place.

Lastly, promoting fiscal autonomy is crucial. Central governments should work towards granting local governments the ability to administer their own taxes and make independent decisions about how to allocate and spend their revenues. This would involve establishing fair and efficient intergovernmental fiscal transfer systems to ensure that local governments have the resources they need to fulfill their responsibilities. Consideration should also be given to implementing performance-based grant systems. Under these systems, a portion of intergovernmental transfers would be allocated based on performance indicators such as revenue collection efficiency or service delivery quality. By implementing these policies, local governments in Africa could successfully implement fiscal decentralization and enhance its beneficial impacts on local economic development.

### Ethics and consent

Ethical approval and written informed consent were not required.

## Data Availability

No data are associated with this article. Figshare: Checklist for Fiscal decentralization in Africa, 10.6084/m9.figshare.25331896.

## Footnotes

^1^
This relates to the physical production of goods and services, involving the acquisition and integration of factor inputs (such as capital, labour, raw materials, land, technology, or management) to generate the desired end products.

^2^
This pertains to executing budget priorities by strategically planning government programs, defining program objectives, conducting service tenders, outsourcing services to the private sector, overseeing and auditing service delivery, and evaluating the effectiveness of implemented programs.

^3^
Intergovernmental fiscal transfers pertain to the redistribution of financial resources from higher tiers of governance to lower-level tiers, namely from central governments to regional or local governments and they are a key tool for managing the balance of resources and responsibilities between different levels of government ( Thomas, Sevidzem, & Wiykiynyuy, 2022).

^4^
The practice of cadre deployment involves appointing party loyalists to key positions, often not qualified, leading to incompetence, corruption, and political interference ( Shava & Chamisa, 2018).

^5^
The erratic nature of coalition refers to the unstable and unpredictable formation and dissolution of local government ( Khumalo & Netswera, 2020).

^6^
Technologists refers to professionals who specialize in the application of scientific knowledge and technical skills to various fields.

^7^
Synchronous e-learning refers to as real-time e-learning or live online learning. This mode of online learning entails instructors and learners engaging in learning activities simultaneously ( Granda, Nuño, Suárez, & Pérez, 2013).
