# Supplementary material for: Digital Tools for Boosting the Impact of Fiscal Decentralization in Africa's Local Economies
Source: F1000Res. 2024 Apr 16;13:279. [Version 1] doi: 10.12688/f1000research.143922.1 (PMC11170072; doi:10.12688/f1000research.143922.1)
Supplement: Peer review history

# Reviewer report: Reviewer response for version 1

**DOI:** 10.5256/f1000research.157648.r270657

**Published:** 2024-06-12

**Authors:** Felix Oppong

**Affiliations:** 1 World Bank, Washington, DC, USA

**Methodology**

 The methodology applied to the research seem appropriate for this type of work. However, Figure 1 does not mirror the description of the methodology. While the authors initially downloaded papers from 246 sources,  and 14 websites, the paper notes that about 108 sources were relevant. These papers included peer-reviewed academic journals and conference proceedings to dissertations, policy briefs, and digitally accessible materials. Figure 1 in the end shows only 30 studies and 30 reports. If the paper is using stratified random sampling technique (which I think is appropriate), it would be great to include the details in Figure 1 or at least ensure that it reflects the description. Figure 1 also indicates that out of 108 reports assessed, 136 of them were excluded. Authors should correction the chart to mirror the narrative.

**Literature Review**

**Presentation of the literature: **Authors did not follow their own format in the section 3 (background: understanding the potential of fiscal decentralization to drive local economic development in Africa), which gives an indication of how they intend to proceed in the paper as follows: “The four pillars we will discuss include the distribution of expenditure responsibilities, the assignment of revenue sources, the allocation of intergovernmental fiscal transfers or grants, and the regulations surrounding subnational borrowing, budget deficits, and the accrual of subnational debt”. Authors should re-order the literature review section along the lines of the sentence.

**Literature on digital tools for fiscal decentralization vs. title of the paper:**  The literature review is globally adequate. The paper uses current and relevant literature to argue it points while discussing empirical literature and identifying constraints facing selected African countries. Authors must address the following comments.

  

- Section 3 to 3.5 do not have strong connection with challenges with the use of digital technologies  which is central to the paper. In each section discussing the four pillars of fiscal decentralization and challenges of  decentralization, it would be useful create linkages necessitating the need for digital tools.  As it stands, the first eleven pages of the paper seem somewhat disconnected with the title of the paper and does not engage the reader gradually to comprehend the title until section 5.

 

- Country cases in the section 3.5  require referencing.

Section 5: Leveraging digital technology for fiscal decentralization and property taxation in Africa: The paper starts full discussion on digital technology in this section. The section covered important areas such as application of evolving digital technology ( including IA powered solutions), automated processes,  digital platforms, use of big data, etc. These are well written.

  

- The paper should note clearly that many of the digital technologies are evolving even in advanced economies and that African countries require time to roll them out.  Also, some of these digital tools are applied by the central governments as such the systems may already be in place at the center, regional capital or metropolitans. In some cases, there may be need for expansion of the coverage to SNG while in other cases, the central government may not have started applying specific tools due to legal arrangements and cost factors. Discussion of factors that impede rapid deployment of digital tool will be great.  For instance, Telecom agreements are needed for fiber optic cables to be laid or cell Tower to be set up  to enable mobile money platforms to be accessible to SNG. The lack of expansion of critical infrastructure to local levels could hamper the extension of certain digital tools.
- Section 5 explains what technological tools are available. Section 5.3 could benefit from “when” and “how” to use these tool to improve fiscal decentralization for SNGs.

**Conclusion**

- The paper draws relevant conclusions. Countries are at different stages of development and may not be in the possible to quickly put in place a provision of robust digital infrastructure. That said, many African Countries beginning to deploy digital tools for revenue collection at the level of Central government. Thus, the conclusion and policy recommendation are appropriate.

Are the rationale for, and objectives of, the Systematic Review clearly stated?

Yes

Is the statistical analysis and its interpretation appropriate?

Not applicable

If this is a Living Systematic Review, is the ‘living’ method appropriate and is the search schedule clearly defined and justified? (‘Living Systematic Review’ or a variation of this term should be included in the title.)

Not applicable

Are sufficient details of the methods and analysis provided to allow replication by others?

Yes

Are the conclusions drawn adequately supported by the results presented in the review?

Yes

Reviewer Expertise:

Fiscal decentralization and taxation.

I confirm that I have read this submission and believe that I have an appropriate level of expertise to confirm that it is of an acceptable scientific standard.

# Reviewer report: Reviewer response for version 1

**DOI:** 10.5256/f1000research.157648.r270659

**Published:** 2024-05-31

**Authors:** Jamie Boex

**Affiliations:** 1 Local Public Sector Alliance, Washington, USA

The topic of the study is an important one, and the initial draft provides a good foundation for a peer-reviewed and approved article. The draft can benefit from revision in a number of different ways.

 1. Introduction 

 The introductory paragraph seems to go into too much detail of the four pillars, rather than setting the scene for the paper. 

 It is a priori unclear to what extent the focus is on the role of IT and all four pillars, or whether the focus is exclusively revenue decentralization, and/or specific LED functions.

 2.Methods

 This sections adds little value, beyond stating that you did a thorough literature review.

 3. FD and potential for LED 

 This section leads the reader to think about LED, but really the section is about the assignment of functions and expenditure responsibilities.

 4. FD challenges

 The section title is misleading.

 Other than RSA and Kenya, weak political and admin decentralization are the #1 and #2 reasons for weak FD in Africa. I suggest you lead with that.

 There should be a discussion on the negative impact of IGFT on OSR incentives and performance.

 Section 5. Leveraging digital technology

 Too much emphasis is given to AI, Machine learning and block chain. The benefits of digital technology--including the ability of IT to improve service delivery processes and offer better value for money in public service delivery; greater transparency; and more effective revenue collection-- could be presented in a clearer and more structured manner, with consistent, concrete examples.

 Section 6. Conclusions

 The minimum efficient scale of digital solutions is high. National governments in Africa are increasingly using digital platforms, but individual LGs do not have the scale to efficiently develop (or purchase) their own built-from-scratch IT solutions.  Instead, they should "contract-in" skills and services from IT firms based on existing platforms (think: parking apps, online platforms for paying registration fees, for issuing local permits,  etc.). Unfortunately, the market for municipal IT services in Africa is still small, because most LGs still work in a traditional (paper-based) public sector manner, and not in a results-based, evidence-based, client-oriented  manner.

 Given that there is currently no market for local digital solutions, the emergence of a market for local digital solution will require simultaneous  action on both the demand and supply. Such systems change is difficult to accomplish. The paper omits any discussion on how to achieve the systems-change needed for digital solutions to become accessible to LGs.

 Overall, the language in the paper should be more nuanced. Statements about the benefits of fiscal decentralization should be qualified (e.g., "Assuming political and administrative decentralization are effective, ...".

Are the rationale for, and objectives of, the Systematic Review clearly stated?

Yes

Is the statistical analysis and its interpretation appropriate?

Partly

If this is a Living Systematic Review, is the ‘living’ method appropriate and is the search schedule clearly defined and justified? (‘Living Systematic Review’ or a variation of this term should be included in the title.)

Not applicable

Are sufficient details of the methods and analysis provided to allow replication by others?

Yes

Are the conclusions drawn adequately supported by the results presented in the review?

Partly

Reviewer Expertise:

Public finance, intergovernmental finance, local government finance, local administration

I confirm that I have read this submission and believe that I have an appropriate level of expertise to confirm that it is of an acceptable scientific standard, however I have significant reservations, as outlined above.

# Reviewer report: Reviewer response for version 1

**DOI:** 10.5256/f1000research.157648.r270654

**Published:** 2024-05-29

**Authors:** François Vaillancourt

**Affiliations:** 1 Universite de Montreal, Montreal, Québec, Canada

This article nicely summarizes the literature on the classical pillars of the decentralization literature with example mainly from Africa. It uses an up to date systematic review of the literature with its methodology described in the methods part of the paper. It  then discusses  the potential of fiscal decentralization to drive local economic development in Africa. Here we would argue that the discussion of borrowing/debt is incomplete. Payment arrears are one way that local governments take on debt. Such arrears vary in kind between countries (wages, social security funds/superannuation, electricity /street lighting , suppliers). Each kind has different consequences. For example, supplier arrears can lead to private suppliers withdrawing supply or charging higher prices (implicit risk premium and interest charge)to compensate for late /uncertain payment. Electricity arrears ,avoided when prepaid cards are used by the utility ,can lead to cuts in services and thus to the incapacity  to access the digital economy

 Fiscal decentralization challenges impeding local economic development in Africa are then presented. The authors quite correctly note the importance of weak institutional capacity as  a prevalent challenge among subnational governments in Africa. The best financial arrangements will come to naught if there is no capacity to manage resources/provide services. Unfortunately, skilled personnel is often reluctant to work at the subnational level in Africa. Finally, the authors put forward recommendations on the use of digital technology to address a subset of issues linked to  fiscal decentralization and property taxation in Africa. They correctly note that this will work only if the digital infrastructure is in place. Thus, it is odd that the provision of electricity is not mentioned in the paper .While more easily provided  now than twenty years ago  in remote areas though solar panels ,it is still lacking in many parts of Sub-Saharan Africa with 50% of the population without it in  2021. ( https://data.worldbank.org/indicator/EG.ELC.ACCS.ZS?locations=ZG )

 Thus, their specific useful recommendations to maximize local revenue potential by better identifying taxpayers and taxable values apply mainly in larger cities unless local revenues are collected centrally as is done in a fair number of African countries. If they work mainly in larger cities, then their implementation should be accompanied by a strengthening of the equalization dimension of the central government transfers to subnational governments to help those left behind .This would be an improvement on many existing scheme that reward already better off larger/capital cities. Finally let us note that the use of various digital tools to analyze matched data bases presupposes that the various silos of a given department, let alone government, will cooperate; this is often hard to achieve.

 In conclusion we agree with the authors who state that " *This paper contributes a valuable perspective to the existing literature, offering actionable insights and policy recommendations that can guide policymakers, scholars, and practitioners in their concerted efforts to realize the full potential of fiscal decentralization in Africa*."

Are the rationale for, and objectives of, the Systematic Review clearly stated?

Yes

Is the statistical analysis and its interpretation appropriate?

Not applicable

If this is a Living Systematic Review, is the ‘living’ method appropriate and is the search schedule clearly defined and justified? (‘Living Systematic Review’ or a variation of this term should be included in the title.)

Not applicable

Are sufficient details of the methods and analysis provided to allow replication by others?

Yes

Are the conclusions drawn adequately supported by the results presented in the review?

Yes

Reviewer Expertise:

Public economics  in particular

I confirm that I have read this submission and believe that I have an appropriate level of expertise to confirm that it is of an acceptable scientific standard.
